# Mathematical Model of Naive T Cell Division and Survival IL-7 Thresholds

**DOI:** 10.3389/fimmu.2013.00434

**Published:** 2013-12-23

**Authors:** Joseph Reynolds, Mark Coles, Grant Lythe, Carmen Molina-París

**Affiliations:** ^1^Department of Applied Mathematics, School of Mathematics, University of Leeds, Leeds, UK; ^2^Centre for Immunology and Infection, University of York, York, UK

**Keywords:** IL-7, T cell, homeostasis, threshold, IL-7R, mathematical model, thymic output

## Abstract

We develop a mathematical model of the peripheral naive T cell population to study the change in human naive T cell numbers from birth to adulthood, incorporating thymic output and the availability of interleukin-7 (IL-7). The model is formulated as three ordinary differential equations: two describe T cell numbers, in a resting state and progressing through the cell cycle. The third is introduced to describe changes in IL-7 availability. Thymic output is a decreasing function of time, representative of the thymic atrophy observed in aging humans. Each T cell is assumed to possess two interleukin-7 receptor (IL-7R) signaling thresholds: a survival threshold and a second, higher, proliferation threshold. If the IL-7R signaling strength is below its survival threshold, a cell may undergo apoptosis. When the signaling strength is above the survival threshold, but below the proliferation threshold, the cell survives but does not divide. Signaling strength above the proliferation threshold enables entry into cell cycle. Assuming that individual cell thresholds are log-normally distributed, we derive population-average rates for apoptosis and entry into cell cycle. We have analyzed the adiabatic change in homeostasis as thymic output decreases. With a parameter set representative of a healthy individual, the model predicts a unique equilibrium number of T cells. In a parameter range representative of persistent viral or bacterial infection, where naive T cell cycle progression is impaired, a decrease in thymic output may result in the collapse of the naive T cell repertoire.

## 1 Introduction

The number of naive T cells in the periphery is determined by a balance between cell loss (death or differentiation) and cell renewal due to cell division and thymic export (1, 2). In humans, at least, the decline in thymic export occurs mainly in childhood, from about a year of age until 20 years of age, when the number of naive T cells is increasing (3). In adults, the decline in thymic export is much less pronounced but the number of naive T cells is, more or less, constant (4). Survival of the naive T cell population in the periphery depends on both common gamma chain cytokines and weak “tonic” signals induced by recognition of self-peptides by the T cell receptor (TCR) (5, 6). IL-7 is required for the homeostatic expansion of naive CD8^+^ and CD4^+^ T cells in lymphopenic hosts, while naive T cells disappear over a 1-month period upon adoptive transfer into IL-7 deficient (IL-7^−^) hosts (7–9).

Signals from recognition of self-peptides bound to major histocompatibility complex (sp-MHC), and IL-7, promote cell survival. Naive T cell survival is impaired when removing access to one of these signals (10–14). Of interest are the mechanisms by which these signals are regulated, and that result in a stable number of naive T cells throughout the lifetimes of mice and humans. In this paper, we focus on IL-7 as a master regulator of naive T cell survival (15). IL-7 is produced by stromal cells in tissues, including fibroblastic reticular cells, marginal reticular cells, and lymphatic endothelial cells (16). These cells produce very small amounts of IL-7 messenger RNA, consistent with IL-7 protein levels limiting T cell expansion. IL-7 is a heparin-sulfate binding protein, and as such, it will bind extra-cellular matrix surrounding stromal cells. Thus, the interaction between naive T cells and stroma controls their homeostasis (17). Recognition of higher affinity, non-self-peptides by the T cell receptor induces naive T cells to undergo an alternative, IL-7 independent, survival program dependent on IL-2 (18).

Naive CD8^+^ T cell responses depend on the amount of IL-7 cells are exposed to (19). At low IL-7 concentrations (<10^−2^ ng ml^−1^), cell viability was impaired; at higher concentrations (>1 ng ml^−1^) cells were observed to proliferate in response to IL-7. This difference might arise from changes in the strength of the IL-7R induced signal the cell receives. For an individual cell, IL-7R induced signaling must be greater than some threshold to prevent the accumulation of pro-apoptotic proteins. Similarly, IL-7R signaling must be greater than a second, higher, threshold to induce cell division. Heterogeneity at the single cell level in IL-7 signaling thresholds (a property reported to depend on expression of IL-7R), resulted in differential survival and division (19). Although these observations are based on two different CD8^+^ T cell receptor transgenic mice, it is assumed that the key principles regarding T cell survival will be found in the repertoire of naive CD4^+^ and CD8^+^ T cells.

We introduce a deterministic mathematical model of the naive T cell population to study the change in human naive T cell numbers from birth to adulthood. We will assume cell survival depends on the availability of IL-7. We do not include availability of sp-MHC as a variable within the model, but assume sp-MHC availability is sufficient to allow cell survival and proliferation, in conjunction with sufficient IL-7 stimulus. We also make the approximation that heterogeneity is constant with changes in age. For a mathematical study of the impact sp-MHC availability has on clonal diversity, the reader is referred to Stirk et al. (20, 21). Our model is a mathematical description of the homeostasis of the naive T cell repertoire, but does not consider stimulation by foreign antigens.

## 2 Materials and Methods

### 2.1 A mathematical description of the size of the peripheral naive T cell population

Stochastic processes provide a method of treating each cell as a distinct, countable object, and permit a more realistic model than a deterministic characterization. Fluctuations in the number of cells can be considered but, in a non-linear stochastic model, approximations are often made to facilitate the analysis. In the linear noise approximation (22), for example, fluctuations are assumed to be of order $\Omega^{\frac{1}{2}}$ for a system of size Ω. The human peripheral T cell compartment is estimated to contain of the order of 10^11^ T cells (3). Letting the system size be the average number of naive T cells in humans, we find 𝒪(Ω) = 10^11^ cells, and correspondingly, fluctuations are expected to be typically 10^5^ − 10^6^ cells in magnitude. That is, we expect fluctuations of approximately 0.001% in the size of the human naive T cell pool due to stochasticity in the per cell division and death rates. Based on these considerations, adopting a deterministic approach to describe the total human peripheral naive T cell population is reasonable.

**Figure 1:**
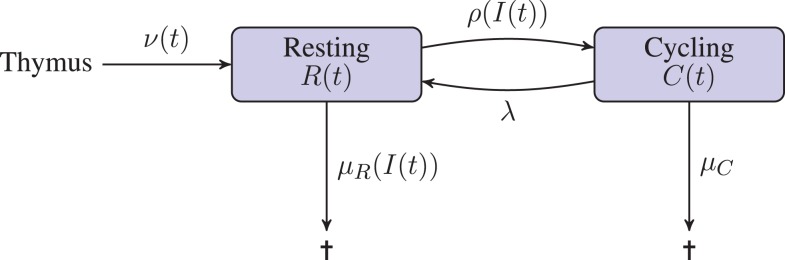
**Diagrammatic illustration of the deterministic model**. T cells leaving the thymus enter the resting naive peripheral pool. Cells in either a resting or cycling state may die. The rate of death from the resting state depends on the availability of the resource (IL-7), whereas the death rate for cycling cells is constant. Resting cells enter the cell cycle at a rate that depends on the availability of the resource (IL-7). Cycling cells produce two daughter cells in the resting state upon completion of the cell cycle.

We assume peripheral naive T cells are either in a resting state, or proceeding through the cell cycle. The deterministic variables *R*(*t*) and *C*(*t*) are introduced to model the total number of T cells in the resting and cycling states, respectively. The variable *I*(*t*) is introduced to model the concentration of IL-7. The deterministic approach we take does not consider any notion of space. Indeed, this approach is tantamount to assuming the resource, IL-7, is shared equally amongst all cells. Competition for the resource is introduced only so far as each cell acts to reduce the global concentration of the resource. Resting cells may receive a signal which induces them to proceed through one round of division. Upon completion of the cell cycle, a cycling cell produces two daughter cells in the resting compartment. Resting cells are assumed to die if the IL-7 induced survival signal is insufficient; cells may also die during cell cycle. The input of cells from the thymus into the resting compartment, in keeping with observations in humans, is a decreasing function of time (23, 24). Production of IL-7 is related to the size of the lymphatic system architecture, which we estimate from the body mass of an individual. In the absence of T cells, IL-7 is assumed to be degraded and/or consumed by other cell types at a constant rate. Upon signal induction through the IL-7 receptor, IL-7 is assumed to be consumed by the T cell. A diagrammatic representation of the model is given in Figure 1.

### 2.2 IL-7 signaling and heterogeneity in IL-7 thresholds

In the model, IL-7 signaling is assumed to be uniform across the population. Yet, we introduce heterogeneity in the signaling thresholds for survival and proliferation. Let *S*(*t*) be the average signaling strength across the naive T cell population. Each cell experiences the same strength of signaling for a given concentration of IL-7, *I*(*t*). We relate the internal signaling to the concentration of IL-7 by the equation

(1) $$S\left(t\right)=\frac{600I\left(t\right)}{0.025+I\left(t\right)}\;.$$

The functional form and constants of this relationship are derived from the study of IL-7 receptor dynamics summarized in the Appendix. We assume each individual cell possesses a unique pair of thresholds for survival and proliferation. Furthermore, we assume, in the continuous limit, that these thresholds are distributed log-normally across the entire population of cells. The use of log-normal distributions guarantees, first of all, that all signaling thresholds are positive real numbers. Secondly, the log-normal distribution ensures that no cell can survive or divide independently of IL-7.

Let the random variable Θ*_s_* represent the survival threshold, and let Θ*_p_* represent the proliferation threshold. We write

(2) $$\begin{array}{llll} \Theta_{s} & ∼\log N\left(\log\theta_{s},\;\frac{1}{2\alpha^{2}}\right)\;,\;\Theta_{p}∼\log N\left(\log\theta_{p},\;\frac{1}{2\alpha^{2}}\right),\; & & \;\\ \alpha & \in R^{+}. \end{array}$$

The respective probability density functions are

(3) $$p_{\Theta_{x}}\left(\theta \right)=\frac{\alpha }{\sqrt{\pi \theta }}\exp\left[-{\left(\alpha \left(\log\theta -\log\theta_{x}\right)\right)}^{2}\right]\;,\;x=s,\;p.$$

Estimates for θ*_s_* and θ*_p_* are obtained from the model summarized in the Appendix. Lauffenburger et al. found a significant change in cell viability for both OT-1 and F-5 T cells at around 10^−2.5^ ng ml^−1^ IL-7 (19). Proliferation of OT-1 cells occurred above 1 ng ml^−1^ IL-7. We estimate the equilibrium signaling at these concentrations as 60 and 600 units, respectively, setting θ*_s_* = 60 and θ*_p_* = 600. Modeling heterogeneity in IL-7 responses by assuming heterogeneity in the IL-7 signaling thresholds, allows us to avoid modeling the naive population using either: (i) a PDE approach, where heterogeneity is continuous across the population of cells, or (ii) describing each subset of cells sharing common thresholds with its unique governing set of ODEs. However, these approaches present an obvious avenue for further research beyond the scope of this paper.

#### 2.2.1 Death rate of resting cells

Suppose each T cell in the naive population is a distinct member possessing a unique signaling threshold for survival. As in the previous section, we assume these signaling thresholds are distributed log-normally (in the continuous limit). We suppose the death rate of an individual cell is Boolean, in the sense that if the global signaling strength is greater than the cell’s individual survival threshold, then the cell can survive indefinitely. Similarly, if the signaling strength is below the survival threshold, the cell will undergo apoptosis at a rate μ*_R_*. The death rate for cell *i* with survival threshold $\theta_{s}^{\left(i\right)}$, is given by

(4) $$\begin{array}{l} f_{s}\left(S\left(t\right),\;\theta_{s}^{\left(i\right)}\right)=\left\{\begin{array}{cc} {\;\mu }_{R}\; & \mathrm{if}\;S\left(t\right)<\theta_{s}^{\left(i\right)}\;,\\ 0\; & \mathrm{if}\;S\left(t\right)\geq \theta_{s}^{\left(i\right)}\;. \end{array}\right. \end{array}$$

In the continuous limit (assuming signaling thresholds are distributed log-normally), the average death rate for the population of naive T cells is given by

(5) $$\begin{array}{llll} {\bar{\mu }}_{R}\left(S\left(t\right)\right) & =\int_{0}^{\infty }\;f_{s}\left(S\left(t\right),\;\theta \right)p_{\Theta_{s}}\left(\theta \right)\;d\theta =\int_{S\left(t\right)}^{\infty }\;\mu_{R}p_{\Theta_{s}}\left(\theta \right)\;d\theta ,\; & & \;\\ & =\frac{1}{2}\mu_{R}\left[1-\mathrm{erf}\left(\alpha \left(\log S\left(t\right)-\log\theta_{s}\right)\right)\right]\;, \end{array}$$

where $p_{\Theta_{s}}\left(\theta \right)$ is the probability density function of the random variable Θ*_s_*, defined by equation (3) with *x* = *s*.

#### 2.2.2 Rate of entry into cell cycle

Analogous to Section 2.2.1, we assume each T cell in the naive population is a distinct member possessing a unique signaling threshold for proliferation. We let the individual rate of entry into cell cycle be given by

(6) $$\begin{array}{l} f_{p}\left(S\left(t\right),\;\theta_{p}^{\left(i\right)}\right)=\left\{\begin{array}{cc} 0\; & \mathrm{if}\;S\left(t\right)<\theta_{p}^{\left(i\right)}\;,\\ \rho \; & \mathrm{if}\;S\left(t\right)\geq \theta_{p}^{\left(i\right)}\;. \end{array}\right. \end{array}$$

Assume, in the continuous limit, the signaling threshold for entry into the cell cycle is represented by the random variable Θ*_p_*, defined in equation (2), with probability density function $p_{\Theta_{p}}\left(\theta \right)$ (equation (3), *x* = *p*). The average rate of entry into cell cycle is given by

(7) $$\begin{array}{llll} \bar{\rho }\left(S\left(t\right)\right) & =\int_{0}^{\infty }\;f_{p}\left(S\left(t\right),\;\theta \right)p_{\Theta_{p}}\left(\theta \right)\;d\theta =\int_{0}^{S\left(t\right)}\;\rho p_{\Theta_{p}}\left(\theta \right)\;d\theta \;,\; & & \;\\ & =\frac{1}{2}\rho \left[1+\mathrm{erf}\left(\alpha \left(\log S\left(t\right)-\log\theta_{p}\right)\right)\right]\;. \end{array}$$

#### 2.2.3 Cell cycle progression

Cycling cells take on average λ^−1^ days to complete the cell cycle. After a cell divides, both daughter cells are produced in the resting state and require a second signal before they can progress through another round of cell division. Cell cycle may be interrupted resulting in the death of the cell. Such death events occur at a rate μ*_C_*.

#### 2.2.4 Thymic export

**Figure 2:**
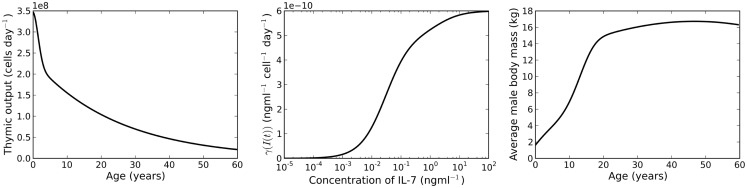
**Left panel: rate of thymic export**. Plot of equation (8). Middle panel: IL-7 internalization rate. Plot of the function γ(*I*(*t*). Right panel: plot of the function *m*(*s*) (equation (11)), a model for the average body mass of male individuals.

We assume thymic output to be a decreasing function of time. In particular, we use the functional form given by Bains et al. (3). Let us introduce the thymic output function, *v*(*t*), as follows

(8) $$\begin{array}{llll} \nu \left(t\right) & =2.32\times 10^{8}\exp\left(-1.1\times 10^{4}t\right)\; & & \;\\ & \;+\;1.15\times 10^{8}\exp\left(-1.6\times 10^{7}t^{2}\right)\;, \end{array}$$

where *t* corresponds to the age of the individual, measured in days. A plot of this function is shown in the left panel of Figure 2. The function was chosen by Bains et al. to describe the rate of thymic export of CD4^+^ T cells. We use the same function to describe the export rate of all naive T cells (CD4^+^ or CD8^+^ T cells). This approximation is justified since we require the absolute cell count to roughly approximate the cell count observed in humans (indeed, such an observation is likely subject to large differences). Of interest later in the paper is the relative variation of cell numbers with different choices of parameter values. For our purposes, the important feature of *v*(*t*) is that it is a decreasing function of time.

#### 2.2.5 Internalization of IL-7

We use the model summarized in the Appendix to estimate the rate of IL-7 internalization. The total number of IL-7 molecules internalized by a single T cell in 1 day, exposed to IL-7 at concentration *I* ng ml^−1^, is described by the function

(9) $$\frac{6.7I\left(t\right)}{2+I\left(t\right)}\;\left(3+\frac{5}{I\left(t\right)+3\times 10^{-2}}\right)\times 10^{3}\;{\mathrm{cell}}^{-1}\;{\mathrm{day}}^{-1}\;.$$

It is reported IL-7 has a molecular mass of 17 kDa (≈2.8 × 10^−11^ ng) (25). Based on this, we define the per cell rate of IL-7 internalization to be

$$\frac{2I\left(t\right)}{2+I\left(t\right)}\;\left(3+\frac{5}{I\left(t\right)+3\times 10^{-2}}\right)\times 10^{-7}\;\mathrm{ng}\;{\mathrm{day}}^{-1}\;{\mathrm{cell}}^{-1}\;.$$

In order to convert the rate of change of mass to the rate of change of concentration, we must choose a volume for the system. Naive T cells are typically found in the lymph nodes, spleen, and gut of the human body. We shall make the rough estimation that the total volume is of the order of 1 l. This implies the rate of IL-7 internalization by all naive T cells in the population is given by

(10) $$\begin{array}{llll} \gamma \left(I\left(t\right)\right)R\left(t\right) & =\frac{2I\left(t\right)}{2+I\left(t\right)}\;\left(3+\frac{5}{I\left(t\right)+3\times 10^{-2}}\right)\; & & \;\\ & \;\times 10^{-10}R\left(t\right)\;{\text{ng ml}}^{-1}\;{\mathrm{day}}^{-1}, \end{array}$$

where *R*(*t*) is the number of resting naive T cells. The IL-7 internalization rate, γ(*I*(*t*), is shown in the middle panel of Figure 2.

#### 2.2.6 Production of IL-7

We assume the rate of IL-7 production is proportional (with proportionality constant $\hat{\beta }$) to the body mass of an individual. To estimate average body mass we use the model given by Burmaster and Crouch (26). The explicit relationship between mass and age is given by the function *m*(*s*) as follows

(11) $$\begin{array}{llll} m\left(s\right) & =\exp\left[4.1+1.4\times 10^{-2}s-1.5\times 10^{-4}s^{2}\right.\; & & \;\\ & \;\left.-2.0\exp\left[-s\left(0.15-1.4\times 10^{-2}s+9.8\times 10^{-4}s^{2}\right)\right]\right]\;, \end{array}$$

where *s* is age measured in years. A plot of this function is given in the right panel of Figure 2. The rate of IL-7 production is given by the function

(12) $$\beta \left(t\right)=\hat{\beta }m\left(365t\right),$$

where *t* denotes age as measured in days.

#### 2.2.7 Intra-cellular degradation of IL-7

We assume IL-7 is degraded and internalized by other cell types at a constant rate. We let the degradation rate of IL-7 be denoted by the parameter δ.

#### 2.2.8 Deterministic mathematical model

From the above assumptions, the system of differential equations governing the behavior of the naive T cells (resting and cycling) and the concentration of IL-7 is given by

(13) $$\begin{array}{ll} \frac{dI\left(t\right)}{dt} & =\beta \left(t\right)-\gamma \left(I\left(t\right)\right)R\left(t\right)-\delta I\left(t\right)\;, \end{array}$$

(14) $$\begin{array}{ll} \frac{dR\left(t\right)}{dt} & =\nu \left(t\right)-\left[\bar{\rho }\left(S\left(t\right)\right)+{\bar{\mu }}_{R}\left(S\left(t\right)\right)\right]R\left(t\right)+2\lambda C\left(t\right)\;, \end{array}$$

(15) $$\begin{array}{ll} \frac{dC\left(t\right)}{dt} & =\bar{\rho }\left(S\left(t\right)\right)R\left(t\right)-\left(\mu_{C}+\lambda \right)C\left(t\right)\;. \end{array}$$

This system is subject to the initial conditions *I*_0_, *R*_0_, and *C*_0_.

## 3 Results

### 3.1 Parameter estimates

The function describing the rate of internalization of IL-7, equation (10), and the signaling relation, equation (1), were both estimated from our studies of IL-7 receptor dynamics in naive T cells (summarized in the Appendix). In the same study we have estimated the average signaling thresholds to be, respectively, θ*_s_* = 60 and θ*_p_* = 600. Our estimate for θ*_p_* was found to be close (θ*_p_* ≈ 585, which we have rounded to 600) to the limit of the signaling function equation (1), as *I*(*t*) → +∞. These estimates imply that, on average, approximately half of the naive T cell population does not proliferate in response to excess IL-7 when receptor dynamics is in equilibrium. Such a finding is consistent with the observations made by Lauffenburger et al. in the experiments described in Ref. (19). In these experiments, F-5 T cells did not proliferate (whereas OT-1 cells did proliferate), even in excess amounts of IL-7. Estimates for the IL-7 average signaling thresholds, θ*_s_* and θ*_p_*, have been based on OT-1 cells. However, we note that changes to these thresholds only result in quantitative differences, provided θ*_s_* < θ*_p_*.

We assume cycling naive T cells take 12 h to complete the cell cycle and produce two daughter cells (λ^−1^ = 0.5 day). Resting naive T cells are assumed to die after 2 days of IL-7 starvation $\left(\mu_{R}^{-1}=2\;\mathrm{day}\right)$. We, furthermore, set the rate of entry into cell cycle to be ρ = 5 day^−1^. Cycling cells are assumed to die at rate μ*_C_* = 1 day^−1^ in healthy individuals, whereas we choose μ*_C_* = 3 day^−1^ (>λ) to be representative of cell cycle impairment. These parameters have been estimated from the literature. We note that the model behavior we discuss in the following sections was found to be robust to changes in these parameters. Robustness was concluded since a 10-fold change in each or any combination thereof, of these four parameters did not change the qualitative behavior of the model, provided the relation λ > μ*_C_* (or λ < μ*_C_*) was maintained. We examine the changes in model behavior arising from altering the relative values of λ and μ*_C_* in the following sections.

We choose δ such that δ*I*(*t*) is similar in magnitude to γ(*I*(*t*)*R*(*t*), when *R*(*t*) ≈ 10^11^, and *I*(*t*) ≈ 10^−2^ ng ml^−1^. The proportionality constant $\hat{\beta }$ is chosen such that we observe 𝒪(10^11^) naive T cells in equilibrium at 20 years of age (for *I*(*t*) ≈ 10^−2^ ng ml^−1^). We had the least inclination when choosing the parameter α. This parameter describes the spread in the individual signaling thresholds across the naive T cell population. We choose α = 2, however this choice has no justification from the literature. The parameter set is summarized in Table 1.

**Table 1:** **Parameter choices for the mathematical model**.

Parameter	Value	Units
θ*_s_*	60	Signaling units
θ*_p_*	600	Signaling units
α	2	(Log signaling units)^−1^
$\hat{\beta }$	0.2	10^−12^ ml^−1^ day^−1^
δ	500	day^−1^
λ	2	day^−1^
ρ	5	day^−1^
μ*_R_*	0.5	day^−1^
μ*_C_*	1	day^−1^

### 3.2 There exists a unique and steady state when λ > μ*_c_*

Let us suppose changes in thymic output and IL-7 production occur in time scales slower than those of the changes in the number of naive T cells. Under this assumption, we look for adiabatic solutions of the system as follows:

(16) $$\begin{array}{ll} 0 & =\beta \left(t\right)-\gamma \left(\hat{I}\left(t\right)\right)\hat{R}\left(t\right)-\delta \hat{I}\left(t\right)\;, \end{array}$$

(17) $$\begin{array}{ll} 0 & =\nu \left(t\right)-\left(\bar{\rho }\left(\hat{S}\left(t\right)\right)+{\bar{\mu }}_{R}\left(\hat{S}\left(t\right)\right)\right)\hat{R}\left(t\right)+2\lambda \hat{C}\left(t\right)\;, \end{array}$$

(18) $$\begin{array}{ll} 0 & =\bar{\rho }\left(\hat{S}\left(t\right)\right)\hat{R}\left(t\right)-\left(\mu_{C}+\lambda \right)\hat{C}\left(t\right)\;. \end{array}$$

**Figure 3:**
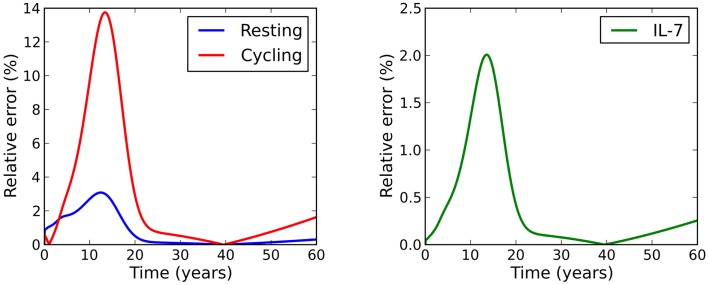
**Relative error between the adiabatic solution and the exact solution, where initial conditions were chosen to be equal to the adiabatic solution at time *t* = 0**.

For the parameter set studied, the relative error between this solution and the exact solution is within 3% for the resting naive T cell population and the concentration of IL-7, and within 14% for the cycling T cell population (see Figure 3).

All numerical results presented in the paper have been obtained with a Python code^1^: differential equations (13), (14), and (15) have been solved using a fourth-order Runge-Kutta scheme. Quasi-stationary solutions were found using the scipy.optimize package. Bifurcation plots were computed using a bisection scheme to search for multiple solutions of equation (21) in the interval [0,1]. Corresponding T cell numbers were calculated using equations (19) and (20).

Notice that the adiabatic solution for both cell types is uniquely defined for a given value of cytokine concentration, $\hat{I}\left(t\right)$, namely

(19) $$\begin{array}{ll} \hat{R}\left(t\right) & =\frac{\beta \left(t\right)-\delta \hat{I}\left(t\right)}{\gamma \left(\hat{I}\left(t\right)\right)}\;, \end{array}$$

(20) $$\begin{array}{ll} \hat{C}\left(t\right) & =\frac{\bar{\rho }\left(\hat{S}\left(t\right)\right)}{\mu_{C}+\lambda }\frac{\beta \left(t\right)-\delta \hat{I}\left(t\right)}{\gamma \left(\hat{I}\left(t\right)\right)}\;. \end{array}$$

The problem of finding adiabatic solutions can then be reduced to finding solutions, $\hat{I}\left(t\right)$, to the one-dimensional equation

(21) $$\frac{\nu \left(t\right)\gamma \left(\hat{I}\left(t\right)\right)}{\beta \left(t\right)-\delta \hat{I}\left(t\right)}={\bar{\mu }}_{R}\left(\hat{S}\left(t\right)\right)+\left(1-\frac{2\lambda }{\lambda +\mu_{C}}\right)\bar{\rho }\left(\hat{S}\left(t\right)\right)\;.$$

**Figure 4:**
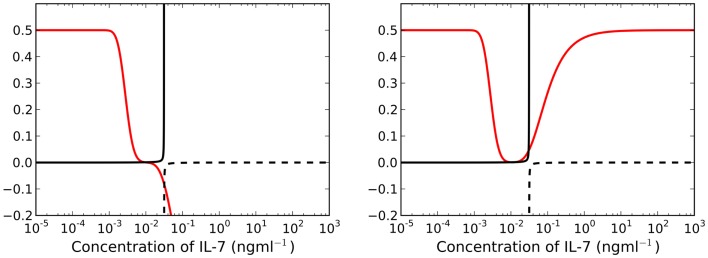
**Left panel: for λ > μ*_C_* (*t* = 25 years), the right-hand side of equation (21) is a decreasing function of *I*(*t*) (red line)**. There exists a unique, asymptotically stable solution $\hat{I}\left(t\right)$ found at the intersection of the solid black and red lines. Right panel: there exist three intersections between the red and solid black lines when λ < μ*_C_* for *t* = 25 years. We require $\beta \left(t\right)>\gamma \left(\hat{I}\left(t\right)\right)$ for existence of stable solutions, therefore we neglect all intersections with the dashed black line.

**Figure 5:**
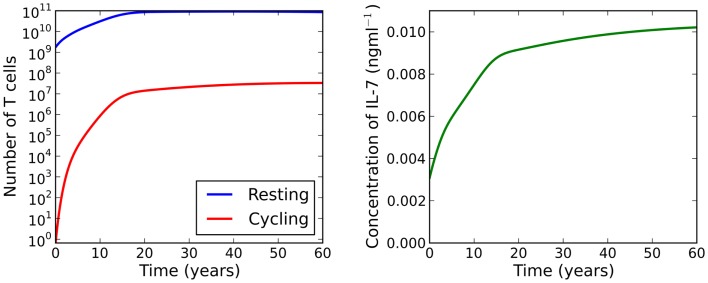
**Numerical solutions to equations (13–15) for λ > μ*_C_***. Initial conditions are chosen to be the adiabatic solutions of equations (16–18) at *t* = 0. We observe a large increase in the naive T cell population from birth to adulthood. During this period we see a 10^7^-fold increase in the number of cycling cells, resulting from increased IL-7 availability and reduced thymic output. IL-7 availability increases continually as the individual ages.

By construction, $\gamma \left(\hat{I}\left(t\right)\right)$ is a monotonically increasing function of $\hat{I}\left(t\right)$. Furthermore, the existence of positive adiabatic solutions requires $\beta \left(t\right)>\delta \hat{I}\left(t\right)$. Therefore, for a fixed time *t*, the left-hand side of equation (21) is an increasing function of $\hat{I}\left(t\right)$. For λ > μ*_C_*, the right-hand side of equation (21) is a monotonically decreasing function of $\hat{I}\left(t\right)$. Lastly, $\gamma \left(\hat{I}\left(t\right)\right)=0$ for $\hat{I}\left(t\right)=0$, and the limit as $\hat{I}\left(t\right)\to 0$ of the right-hand side is equal to μ*_R_*. It follows that the intersection of the left and right sides must be unique and positive. We deduce that for λ > μ*_C_* there exists a unique adiabatic solution to the system (see left plot of Figure 4). This solution is stable for the parameter set given in Table 1. We have also numerically explored parameter space, but have not found a parameter set for which this solution is unstable. In Figure 5 we present numerical solutions to equations (13–15), computed with a fourth-order Runge-Kutta method implemented in Python. Initial conditions were chosen to be the adiabatic solutions of equations (16–18) at *t* = 0.

### 3.3 There exist two steady states when λ < μ*_C_*

**Figure 6:**
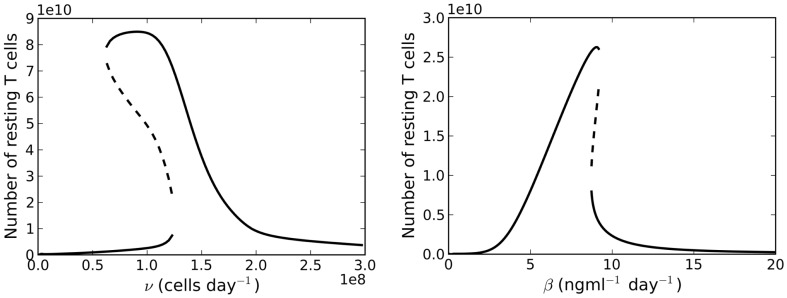
**Left panel: bifurcation diagram for varying the parameter *v*(*t*) for λ < μ*_C_***. Right panel: bifurcation plot as a function of varying the rate of IL-7 production. Thymic export is fixed at *v*(60 years).

Let us now consider the case λ < μ*_C_*. The right-hand side of equation (21) is no longer a decreasing function of $\hat{I}\left(t\right)$. We find there may exist up to three solutions $\hat{I}\left(t\right)$, two of which may be stable simultaneously, whilst the third is unstable (see right plot of Figure 4). Further examination (by numerically finding all solutions using the bisection method) of the model reveals a saddle-node bifurcation as thymic output changes with age (see left panel, Figure 6). We assume that individuals with (around) 10^11^ naive T cells are healthy, in the sense that they have a sufficient number of T cells to provide protection against immune challenges. When a cell in cycle is more likely to die than to produce two daughter cells, we define cell cycle progression to be impaired. Mathematically, this corresponds to λ < μ*_C_*. When cell cycle progression is impaired, the model predicts an individual may possess a healthy number of T cells, thereby being immuno-competent, up until the age at which the model bifurcates. For the parameter set we have investigated, this bifurcation is inevitable given the estimated decline in thymic output established by Bains et al. (3). Indeed, for a given parameter set, from the known rate at which thymic output declines, one can estimate the age at which the model bifurcates. The bifurcation results in a decrease in the naive T cell population size of approximately two orders of magnitude, that is, following the bifurcation we expect roughly 99 out of every 100 naive T cells to be lost.

## 4 Discussion

In a healthy individual, it is reasonable to expect that naive T cells entering the cell cycle are more likely to complete division and produce two daughter cells, than to die during the division process. Therefore, we suppose the parameter relation λ < μ*_C_* represents a healthy individual. We have shown, under this hypothesis, that the model allows a single asymptotically stable adiabatic solution. The total number of naive T cells per kilogram of body mass was found to increase over the first 18 years of life and decrease thereafter. The decline in this ratio for adults is seemingly a consequence of the reduction in thymic output.

The number of cycling naive T cells in adiabatic conditions was found to increase from practically 0 (approximately 0.75 cells) cells at birth to 10^7^ cells by adulthood. Whilst the number of cycling cells increased thereafter, the increase was at a markedly slower rate. The increase in the number of cycling cells is probably due to the decline in thymic output, wherein competition for IL-7 decreases. Despite the increase in the cycling population, the number of cells in cycle is several orders of magnitude smaller than the resting population. For adults, this proportion is approximately [0.01, 0.04]% of the total naive population.

**Figure 7:**
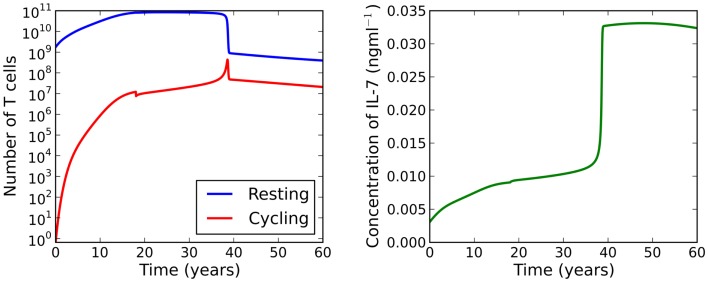
**Theoretical scenario in which we set μ*_C_* = 3 at 18 years of age**. At roughly 33 years of age the model bifurcates resulting in a dramatic loss of naive T cells.

When cell cycle is impaired (λ < μ*_C_*), the model exhibits a saddle-node bifurcation as thymic output declines. For the parameter set investigated, the two adiabatic solutions are generally separated by two orders of magnitude. This feature motivates the following theoretical scenario: suppose a healthy individual experiences some event which causes naive cell cycle progression to become impaired at 18 years of age. The model predicts the naive T cell repertoire will experience a dramatic reduction in cell numbers at roughly 33 years of age. See Figure 7 for the full solution of the model illustrating this scenario. From an immunological perspective, this scenario is interesting because the noticeable effect of the event (the dramatic loss of naive T cells) occurs roughly 15 years later than the event itself (naive cell cycle progression to become impaired at 18 years of age). Indeed, it is the reduction in thymic output that triggers the loss rather than the event itself. If the thymus was not atrophic, the loss in naive T cells would not occur.

**Figure 8:**
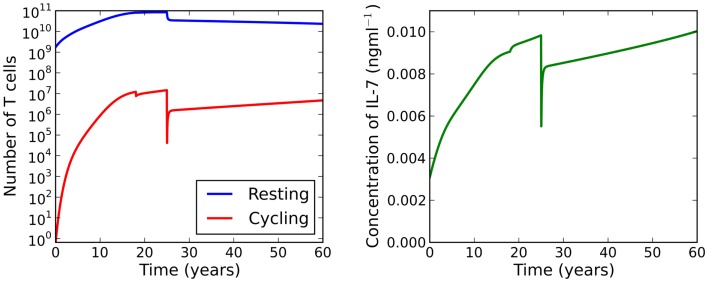
**At age 18 the individual experiences some event resulting in impaired cell cycle progression**. At age 25 the individual undergoes treatment to limit IL-7 production to levels comparable to those at 11 years of age. Whilst the T cell count is reduced, this attrition is a significant improvement on the reduction observed when the individual has not received treatment (see Figure 7).

Suppose now we fix thymic output at a constant rate and let the cell cycle be impaired. The model undergoes a saddle-node bifurcation as the rate of IL-7 production changes. More specifically, the model bifurcates, resulting in a decrease in the total number of T cells, as a consequence of increasing IL-7 production. Intuitively, this can be understood as follows: for increased IL-7 production, the rate of entry into cell cycle is enhanced, however, since cells are more likely to die in cell cycle than to produce two daughter cells, the enhanced rate of entry into cell cycle actually serves to decrease the total amount of naive T cells. The bifurcation diagram for this scenario is shown in the right panel of Figure 6. We found the critical value of this bifurcation decreases with thymic output. In the limiting case, corresponding to thymic output at 60 years of age, we found this critical value to be approximately 9.1 ng ml^−1^. Consider again the theoretical scenario in which a healthy individual undergoes an event resulting in cell cycle impairment at 18 years of age. Suppose now at age 25 the same individual undergoes some treatment to limit IL-7 production to 8 ng ml^−1^, corresponding to thymic production at approximately 11 years of age. The T cell count is reduced by approximately 60%. This reduction is a significant improvement on the 99% T cell loss in the untreated individual at 33 years of age. For this theoretical scenario, the model predicts limiting IL-7 availability will partially avoid the dramatic T cell loss arising from reducing thymic export when the cell cycle is impaired. See Figure 8 for the full model solution in the treated individual.

In the model presented here we have neglected the fact naive T cells become activated in response to recognition of ligand specific to their unique TCR. Research by Koenen et al. has shown T cell survival is IL-7 independent following T cell activation (18). Suppose now we include a term in the governing ODEs to represent differentiation of naive T cells into cells with a different phenotype, such as activated T cells. Assuming no reversion back to the naive phenotype, such a term would appear in the model as a loss term equivalent to the death rate ${\hat{\mu }}_{R}\left(S\left(t\right)\right)$. The simplest approach to including differentiation (due to activation) would be to assume naive T cells differentiate into activated T cells at a constant rate proportional to the rate of antigenic challenge. In this case, we would replace the term ${\hat{\mu }}_{R}\left(S\left(t\right)\right)$ by ${\hat{\mu }}_{R}\left(S\left(t\right)\right)+\mu_{D}$, where μ*_D_* is constant. Consider again the red curves in Figure 4. The differentiation term will cause a translation in the red curve of length μ*_D_* up the vertical axis. For λ > μ*_C_*, there still exists a unique solution, however there will be a quantitative change in comparison to the case when μ*_D_* = 0. When λ < μ*_C_*, there still exists the possibility of a saddle-node bifurcation for a general parameter set. For the parameter set we have investigated, there exists a maximum value $\mu_{D}^{*}$, for a given time *t*, such that we can find more than one solution. For differentiation rates $\mu_{D}>\mu_{D}^{*}$, we can only find a unique solution corresponding to the stable adiabatic one, in which we have reduced T cell numbers.

In this paper we have developed and analyzed a deterministic mathematical model of a population of naive T cells, whose survival depends on the availability of the cytokine IL-7. We have shown this model predicts a stable population of cells when cell cycle progression is healthy. More interestingly, when cell cycle progression is impaired, our results indicate declining thymic output may result in a dramatic loss in the number of naive T cells. Furthermore, we have been able to establish that limiting IL-7 production partially rescues this decline. However, our study has been restricted to naive T cells and we have not taken into account the accumulation of memory T cells as an individual ages. Memory T cells are generated in response to antigen or homeostatic cytokines (27), and during repeated homeostasis-driven divisions of naive T cells (28). Previous studies have shown the percentage of memory T cells increases with age, yet the percentage of naive T cells decreases with age (29). CD4^+^ memory T cells also require IL-7 for survival and proliferation in the periphery (30). In the case of CD8^+^ memory T cells, IL-15 is largely responsible for their peripheral survival, but IL-7 may also be required. It is, then, reasonable to assume that memory T cells compete for the IL-7 required for naive T cell survival. At least, that is, for those memory T cells which access IL-7 in the same tissues as naive T cells, such as the lymph nodes. As mentioned before, memory T cells can be derived from repeated divisions of naive T cells (31). In our model, the naive T cells that will acquire a memory-like phenotype first are those with low IL-7 survival and proliferation thresholds. We would expect, then, that over time the ratio of naive to memory T cells would decrease. Furthermore, the naive population would lose those cells with lowest survival and division thresholds. Given the distribution of signaling thresholds in our model, we would expect to see a shift to the right for the distribution of both survival and signaling thresholds. This shift, which implies that on average naive T cells require a higher concentration of IL-7 to enter cell cycle with age, together with the additional competition from an increasing memory population, is expected to cause a decrease in the total number of naive T cells and the percentage of naive T cells in cell cycle. These considerations might help explain the discrepancy between our model and data showing a reduction in the percentage of naive T cells expressing Ki67^+^ during childhood^2^. There is support for the fact that childhood might be the phase in which most memory T cells are acquired (3). Indeed, it might be that the model presented here, better describes the total number of T cells that require IL-7, naive and memory, rather than just the naive T cell pool alone. This, however, would require a more detailed analysis, out of the scope of this paper.

Many different receptor-mediated signals are integrated by T cells in their micro-environment, such as cytokines, adhesion molecules, T cell receptors, and co-receptors (CD28, CTLA-4). However, the survival of naive T cells, the focus of this paper, has been shown to depend on IL-7 and low level TCR stimulation (8, 9, 15, 32). Other cytokines, such as IL-15, play a significant role in the homeostasis of memory CD8^+^ T cells (33). Given the experimental support for the hypothesis that IL-7 is critical for the homeostatic proliferation and the survival of naive T cells, in this first model we have neglected other signals (7).

Previous mathematical models of naive T cell homeostasis have focused on the relative contribution of thymic export and cell division in the periphery (3, 34, 35). These models conclude that in humans, thymic export makes an important contribution to the size of the naive T cell population in early life (<20 years of age), whereas later in life the number of naive T cells is maintained by homeostatic proliferation in the periphery. Some recent studies have measured the relative contribution of thymic export by examining the average number of TRECs in naive T cells (3, 35, 36). The use of mathematical models has allowed these groups to infer the relative (young versus old) kinetics for naive T cells, based on experimental estimates of the total number of naive T cells and recent thymic emigrants (3, 34, 35). In the model introduced here, we have aimed to provide a mechanistic perspective by investigating at the molecular and cellular levels, the role IL-7 plays in regulating the homeostasis of naive T cells (37). The increase in the proportion of cycling cells in our model is in agreement with previous experimental studies (38). Our study suggests the increase in peripheral division rates can be explained by the availability of IL-7, which is a consequence of a combined effect: (i) an increased net IL-7 production as an individual ages, and (ii) a reduction of recently exported thymocytes competing for this resource.

## Conflict of Interest Statement

The authors declare that the research was conducted in the absence of any commercial or financial relationships that could be construed as a potential conflict of interest.

## Figures and Tables

**Figure A1:**
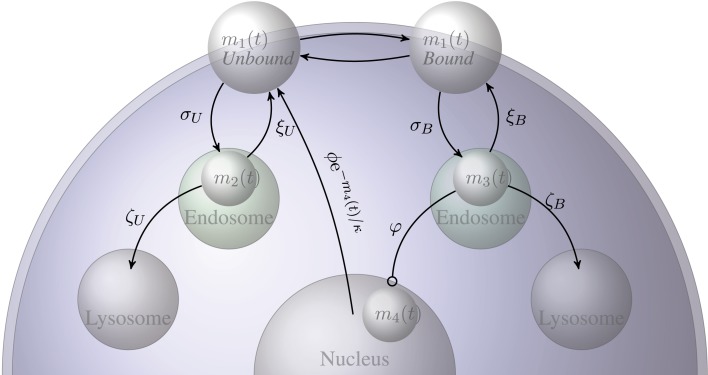
**Diagrammatic representation of the transition probabilities of the stochastic model for the IL-7 and IL-7 receptor system**.

## Footnotes

^1^Python code available upon request.

^2^Our current model suggests the percentage of naive T cells in cell cycle is increasing.

## Appendix

### A.1 A model of IL-7R dynamics

In this section we present a summary of a stochastic model in which we consider the number of IL-7 receptors on the surface of naive T cells. This study is used to derive equations (1) and (9) in the main text. The model has been formulated as a continuous time Markov process. We consider the number of receptors on a single naive T cell. For our purposes, we present the mean field approximation to the model with which we estimate the parameters. We introduce the following four variables:

- *m*_1_(*t*) – the total number of IL-7 receptors on the surface of a naive T cell,
- *m*_2_(*t*) – the number of unbound internalized receptors,
- *m*_3_(*t*) – the number of bound internalized receptors,
- *m*_4_(*t*) – the quantity of IL-7 induced signal.

We assume, in equilibrium, the fraction of surface receptors bound to IL-7 is given by *f_I_*. The system of ODEs denoting the mean field approximation to the stochastic model is given by:

(A1) $$\begin{array}{llll} \frac{dm_{1}\left(t\right)}{dt} & =\phi \;e^{-m_{4}\left(t\right)∕\kappa }+\xi_{U}\;m_{2}\left(t\right)+\xi_{B}\;m_{3}\left(t\right)\; & & \;\\ & \;-\left[\sigma_{U}\left(1-f_{I}\right)+\sigma_{B}f_{I}\right]m_{1}\left(t\right)\;, \end{array}$$

(A2) $$\begin{array}{ll} \frac{dm_{2}\left(t\right)}{dt} & =\sigma_{U}\left(1-f_{I}\right)m_{1}\left(t\right)-\left(\xi_{U}+\zeta_{U}\right)m_{2}\left(t\right)\;, \end{array}$$

(A3) $$\begin{array}{ll} \frac{dm_{3}\left(t\right)}{dt} & =\sigma_{B}f_{I}\;m_{1}\left(t\right)-\left(\xi_{B}+\zeta_{B}\right)m_{3}\left(t\right)\;, \end{array}$$

(A4) $$\begin{array}{ll} \frac{dm_{4}\left(t\right)}{dt} & =\varphi \;m_{3}\left(t\right)-\chi \;m_{4}\left(t\right)\;. \end{array}$$

### A.2 Reduced model in the case when *I* = 0

Consider a T cell in an IL-7 free medium with initial conditions such that the IL-7 induced signaling vanishes and the number of IL-7:IL-7R internal complexes is zero. Then the mean field model can be reduced to the following set of ODEs:

$$\begin{array}{llll} \frac{dm_{1}\left(t\right)}{dt} & =\phi +\xi_{U}\;m_{2}\left(t\right)-\sigma_{U}\;m_{1}\left(t\right)\;,\; & & \;\\ \frac{dm_{2}\left(t\right)}{dt} & =\sigma_{U}\;m_{1}\left(t\right)-\left(\xi_{U}+\zeta_{U}\right)m_{2}\left(t\right).\; & & \; \end{array}$$

This reduced system is governed by four parameters ϕ, ξ*_U_*, σ*_U_*, and ζ*_U_*, and possesses the following stable steady state:

$$\begin{array}{llll} m_{1}^{*} & =\frac{\phi \left(\xi_{U}+\zeta_{U}\right)}{\sigma_{U}\zeta_{U}}\;,\; & & \;\\ m_{2}^{*} & =\frac{\phi }{\zeta_{U}}\;.\; & & \; \end{array}$$

We assume in the reduced model 10% of the total number of receptors are internalized in equilibrium. We set $m_{1}^{*}=9m_{2}^{*}$. Based on the measurements of Singer et al. (39), we shall assume 4 × 10^4^ receptors in total when the reduced model is in steady state. Therefore, we let

(A5) $$\begin{array}{ll} \frac{\phi \left(\xi_{U}+\zeta_{U}\right)}{\sigma_{U}\zeta_{U}} & =3.6\times 10^{4}, \end{array}$$

(A6) $$\begin{array}{ll} \frac{\phi }{\zeta_{U}} & =4\times 10^{3}\;. \end{array}$$

In Ref. (40), cells were cultured with the translation inhibitor cycloheximide (CHX) to prevent transcription of the IL-7 receptor. Total expression of the IL-7 receptor was measured over several time points, from which the authors estimate the half-life of the receptor in an unstimulated cell to be approximately 24 h.

In the reduced model, all receptors are guaranteed to be degraded in a finite amount of time. The expected time for a receptor, that is initially on the cell surface, to be degraded in the lysosome is given by

$$\tau_{1}=\frac{\zeta_{U}+\sigma_{U}+\xi_{U}}{\sigma_{U}\zeta_{U}}\;.$$

Assuming exponential decay, the half-life for a receptor to undergo lysosomal degradation, starting on the cell surface, is then given by

$$t_{\frac{1}{2}}=\frac{\zeta_{U}+\sigma_{U}+\xi_{U}}{\sigma_{U}\zeta_{U}}\log2\;.$$

Thus, we can write

(A7) $$\frac{\zeta_{U}+\sigma_{U}+\xi_{U}}{\sigma_{U}\zeta_{U}}\log2=24\;h\;.$$

Combining equations (A5), (A6), and (A7) we find

$$\begin{array}{llll} \zeta_{U} & \approx 0.29\;h^{-1}\;,\; & & \;\\ \phi & \approx 1.2\times 10^{3}\;\mathrm{receptors}\;h^{-1}\;,\; & & \;\\ \xi_{U}+0.29\;h^{-1} & \approx 9\sigma_{U}.\; & & \; \end{array}$$

The value of ξ*_U_* relative to ζ*_U_* effectively dictates the ratio of receptors which are degraded to those recycled back to the cell surface. We assume the system has evolved to minimize waste of functional proteins and tentatively let ξ*_U_* > ζ*_U_*. That is, we assume that a greater fraction of receptors are recycled back to the surface of the cell. We somewhat arbitrarily set

$$\xi_{U}=1\;h^{-1}\;\Rightarrow \;\sigma_{U}\approx 0.14\;h^{-1}\;.$$

### A.3 Receptor-ligand kinetics

Suppose the number of surface receptors is constant and denoted by *R_T_*. Let us also assume the extra-cellular concentration of IL-7 is constant and denoted by *I*. Define *R_B_*(*t*) to be the number of IL-7 receptors bound to IL-7. Note that we assume the time to recruit the common gamma chain, $\gamma_{c}$, is negligible. Then, we can describe changes in the number of bound complexes by the following ODE

$$\frac{dR_{B}\left(t\right)}{dt}=k_{+}\left[R_{T}-R_{B}\left(t\right)\right]I-k_{-}R_{B}\left(t\right)\;,$$

where *k*_+_ and *k*_−_are, respectively, the binding and unbinding rates of the IL-7:IL-7R receptor-ligand system. We assume the timescales for this reaction are faster than the timescales for changes in receptor numbers, such that we can consider these reactions to be in equilibrium. This ODE has a unique stable steady state:

$$R_{B}^{*}=\frac{R_{T}I}{\frac{k_{-}}{k_{+}}+I}=f_{I}R_{T}\;.$$

The Appendix for Ref. (41) provides estimates for *k*_+_ and *k*_−_, from which we set *k*_+_ = 1 nM min^−1^ and *k*_−_ = 0.1 min^−1^. It is reported IL-7 has a molecular mass of around 17 kDa, from which we estimate the ratio *k*_−_/*k*_+_ ≈ 1.7 ng ml^−1^.

### A.4 Early internalization events

In Ref. (40), surface receptor expression was assessed in human thymocytes. It is reported, cells in 50 ng ml^−1^ IL-7 culture down-regulated IL-7R expression. A 20% reduction was observed after 10 min. Using the above estimate for *k*_−_/*k*_+_, we find *f_I_*|*_I_*_=50_ ≈ 0.97. Based on this, we can neglect internalization of the unbound receptor. In the first 10 min, we shall also neglect recycling and inhibition of receptor transcription. We assume surface receptor expression loss is modeled by the ODE

$$\frac{dm_{1}\left(t\right)}{dt}=\phi -\sigma_{B}m_{1}\left(t\right)\;.$$

Given initial surface expression levels equal to *m*_1_(0), this ODE has solution

(A8) $$m_{1}\left(t\right)=\frac{\phi }{\sigma_{B}}+\left[m_{1}\left(0\right)-\frac{\phi }{\sigma_{B}}\right]e^{\sigma_{B}t}\;.$$

We assume the previous estimates obtained from the reduced model for *m*_1_(0) and ϕ. That is, we let *m*_1_(0) = 3.6 × 10^4^ receptors and ϕ = 1.2 × 10^3^ receptors h^−1^. Then using the above expression for *m*_1_(*t*) equation (A8), we obtain an estimate for σ*_B_*. We find σ*_B_* ≈ 1.4 h^−1^. The authors of Ref. (40) estimate the half-life of the IL-7 receptor in cells treated with CHX, cultured in 50 ng ml^−1^, to be approximately 3 h. The expected time to lysosomal degradation for a surface receptor is given by

$$\begin{array}{llll} \tau_{2} & =\frac{\left[\sigma_{U}\left(1-f_{I}\right)+\left(\xi_{U}+\zeta_{U}\right)\right]\left(\xi_{B}+\zeta_{B}\right)+\sigma_{B}f_{I}\left(\xi_{U}+\zeta_{U}\right)}{\sigma_{U}\left(1-f_{I}\right)\zeta_{U}\left(\xi_{B}+\zeta_{B}\right)+\sigma_{B}f_{I}\left(\xi_{U}+\zeta_{U}\right)\zeta_{B}}\; & & \;\\ & \approx \frac{1.3\;h^{-1}\left(\xi_{B}+\zeta_{B}\right)+1.7\;h^{-2}}{4.6\times 10^{3}\;h^{-2}\left(\xi_{B}+\zeta_{B}\right)+1.7\;h^{-2}\zeta_{B}}.\; & & \; \end{array}$$

Assuming exponential decay, with a half-life of 3 h, we find ξ*_B_* ≈ 0.2ζ*_B_* + 0.3 h^−1^. Again, without a direct measurement, let us set ξ*_B_* = 1 h^−1^, to obtain an estimate for ζ*_B_* ≈ 3.5 h^−1^.

### A.5 Remaining parameters

Consider the observation of an approximately 98% reduction in surface receptor expression following overnight culture in 6 ng ml^−1^ IL-7 made in Ref. (39). We let ${\left.m_{1}^{*}\right|}_{I=6}=0.02\;{\left.m_{1}^{*}\right|}_{I=0}=0.02\frac{\phi \left(\xi_{U}+\zeta_{U}\right)}{\sigma_{U}\zeta_{U}}\approx 763\;\mathrm{receptors}$. We set the derivatives equal to zero in the system of ODEs equations (A1–A4), and manipulate the resulting system of equations to obtain

(A9) $$\begin{array}{llll} & \phi \exp\;\left(-\frac{\varphi \sigma_{B}f_{I}}{\chi \kappa \left(\xi_{B}+\zeta_{B}\right)}m_{1}^{*}\right)\; & & \;\\ & =\left[\sigma_{U}\left(1-f_{I}\right)+\sigma_{B}f_{I}-\frac{\sigma_{U}\left(1-f_{I}\right)\xi_{U}}{\xi_{U}+\zeta_{U}}-\frac{\sigma_{B}f_{I}\xi_{B}}{\xi_{B}+\zeta_{B}}\right]\;m_{1}^{*}\;,\;\\ \Rightarrow \; & \exp\;\left(-1.9\times 10^{2}\;\mathrm{receptors}\;\frac{\varphi }{\chi \kappa }\right)\approx 0.54\;,\; & & \;\\ \Rightarrow \; & \frac{\varphi }{\chi \kappa }\approx 3.2\times 10^{-3}\;{\mathrm{receptors}}^{-1}.\; & & \; \end{array}$$

From the steady solutions of ODEs equations (A3) and (A4), we find

$$m_{4}^{*}=\frac{\phi \sigma_{B}f_{I}}{\left(\xi_{B}+\zeta_{B}\right)\chi }m_{1}^{*}\approx 1.9\times 10^{2}\;\mathrm{receptors}\;\frac{\varphi }{\chi }.$$

We use this expression to rearrange equation (A9) in terms of $m_{4}^{*}$. This gives

$$\exp\left(-\frac{m_{4}^{*}}{\kappa }\right)\approx 9.1\frac{m_{4}^{*}}{\kappa }\;.$$

Solving the above expression numerically, we find $m_{4}^{*}∕\kappa \approx 0.1$. We estimate a value for χ based on the observation that following culture in 6 ng ml^−1^ IL-7, mRNA levels took approximately 12 h to return to 99% of the control levels. The transcription rate is given by ϕexp(−*m*_4_(*t*)/κ). We again assume the IL-7 induced signal decays according to the equation *m*_4_(*t*) = *m*_4_(0)e^−^*^χt^*, where *m*_4_(0) = 0.1. Combing these assumptions with the experimental observations, we have

$$\phi \exp\left[-\frac{m_{4}\left(0\right)\exp\left(-12\chi \right)}{\kappa }\right]=0.99\phi ,$$

from which we find χ ≈ 0.19 h^−1^ ⇒ φ ≈ 6.1 × 10^−4^ receptors^−1^ h^−1^ κ. The parameter κ was chosen to be 1000. This choice was made from the stochastic model: κ = 1000 is the minimum value (to the nearest power of 10) such that fluctuations in the signaling quantity are greater than zero for low (10^−2^ ng ml^−1^) concentrations of IL-7. Using this value for κ, we find ϕ ≈ 0.61 h^−1^. The parameter estimates are summarized in Table A1.

**Table A1:** **Parameter estimates obtained from the mean field model of IL-7 receptor dynamics**.

Parameter	Value	Units
ϕ	1.2 × 10^3^	rec h^−1^
κ	10^3^	sig
ξ*_U_*	1	h^−1^
ξ*_B_*	1	h^−1^
σ*_U_*	0.14	h^−1^
σ*_B_*	1.4	h^−1^
*k*_−_/*k*_+_	1.7	ng ml^−1^
ζ*_U_*	0.29	h^−1^
ζ*_B_*	3.5	h^−1^
φ	0.61	sig rec^−1^ h^−1^
χ	0.19	h^−1^

*We let *sig* be the units of the signaling and *rec* be the units of surface receptor numbers*.

#### A.5.1 Changes in the concentration of IL-7

The functional form

(A10) $$S=\frac{aI}{b+I}$$

is used to approximate the numerical solution of $m_{4}^{*}$ as a function of the concentration of IL-7. Using this function we find *a* ≈ 600 signaling units and *b* ≈ 0.025 ng ml^−1^. In a similar manner we use the functional form

(A11) $$R=c+\frac{d}{e+I}$$

to approximate the numerical solution of $m_{1}^{*}$ as a function of the concentration of IL-7. We find *c* ≈ 600 receptors, *d* ≈ 1000 receptors ng ml^−1^ and *e* ≈ 0.03 ng ml^−1^. The number of IL-7 molecules internalized by each T cell per day is assumed to be the same as the number of internalized IL-7:IL-7R complexes per day. We let the complex internalization rate, as a function of *I*, be given by

(A12) $$\begin{array}{llll} 24\sigma_{B}f_{I}m_{1}^{*}\left(I\right) & \approx \frac{6.7I}{2+I}\left(3+\frac{5}{3\times 10^{-2}+I}\right)\; & & \;\\ & \;\times 10^{3}\;\mathrm{molecules}\;{\mathrm{cell}}^{-1}\;{\mathrm{day}}^{-1}. \end{array}$$
